# Supplementary material for: Role of Klhl14 in senescence and epithelial-to-mesenchymal transition via TGF-β modulation
Source: Cell Death Dis. 2026 Jun 30;17(1):813. doi: 10.1038/s41419-026-09048-7 (PMC13586328; doi:10.1038/s41419-026-09048-7)
Supplement: Supplementary file 1 [file 41419_2026_9048_MOESM1_ESM.pdf]

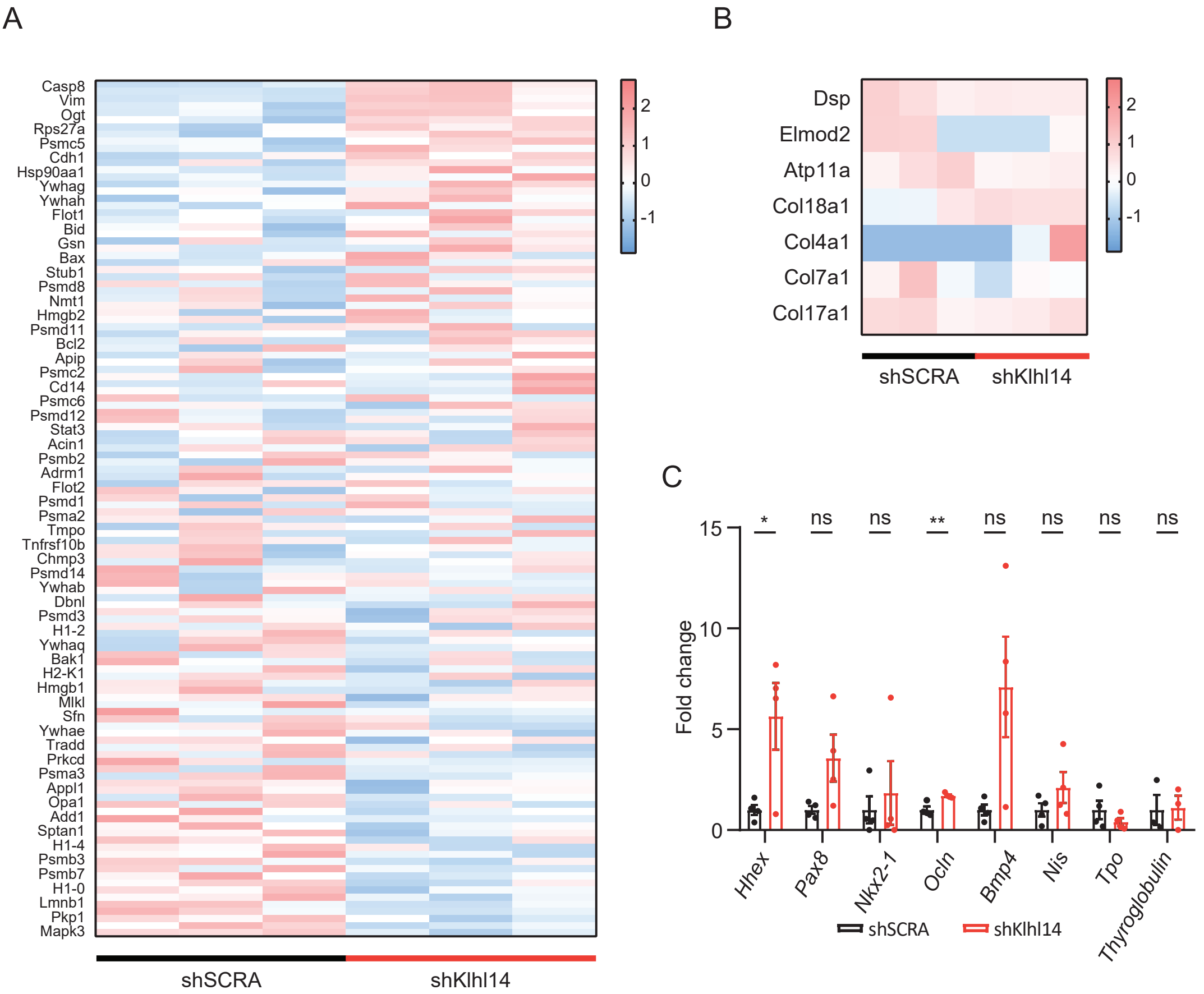

A
Casp8
Vim
Ogt
Rps27a
Psmc5
Cdh1
Hsp90aa1
Ywhag
Ywhah
Flot1
Bid
Gsn
Bax
Stub1
Psmd8
Nmt1
Hmgb2
Psmd11
Bcl2
Apip
Psmc2
Cd14
Psmc6
Psmd12
Stat3
Acin1
Psmb2
Adrm1
Flot2
Psmd1
Psma2
Tmpo
Tnfrsf10b
Chmp3
Psmd14
Ywhab
Dbnl
Psmd3
H1-2
Ywhaq
Bak1
H2-K1
Hmgb1
Mlkl
Sfn
Ywhae
Tradd
Prkcd
Psma3
Appl1
Opa1
Add1
Sptan1
H1-4
Psmb3
Psmb7
H1-0
Lmnb1
Pkp1
Mapk3
2
1
0
-1
shSCRA
shKlhl14
B
Dsp
Elmod2
Atp11a
Col18a1
Col4a1
Col7a1
Col17a1
2
1
0
-1
shSCRA
shKlhl14
C
15
10
5
0
Fold change
*
ns
ns
**
ns
ns
ns
ns
Hhex
Pax8
Nkx2-1
Ocln
Bmp4
Nis
Tpo
Thyroglobulin
shSCRA
shKlhl14
