# Supplementary material for: Role of Klhl14 in senescence and epithelial-to-mesenchymal transition via TGF-β modulation
Source: Cell Death Dis. 2026 Jun 30;17(1):813. doi: 10.1038/s41419-026-09048-7 (PMC13586328; doi:10.1038/s41419-026-09048-7)
Supplement: Supplementary file 2 [file 41419_2026_9048_MOESM2_ESM.pdf]

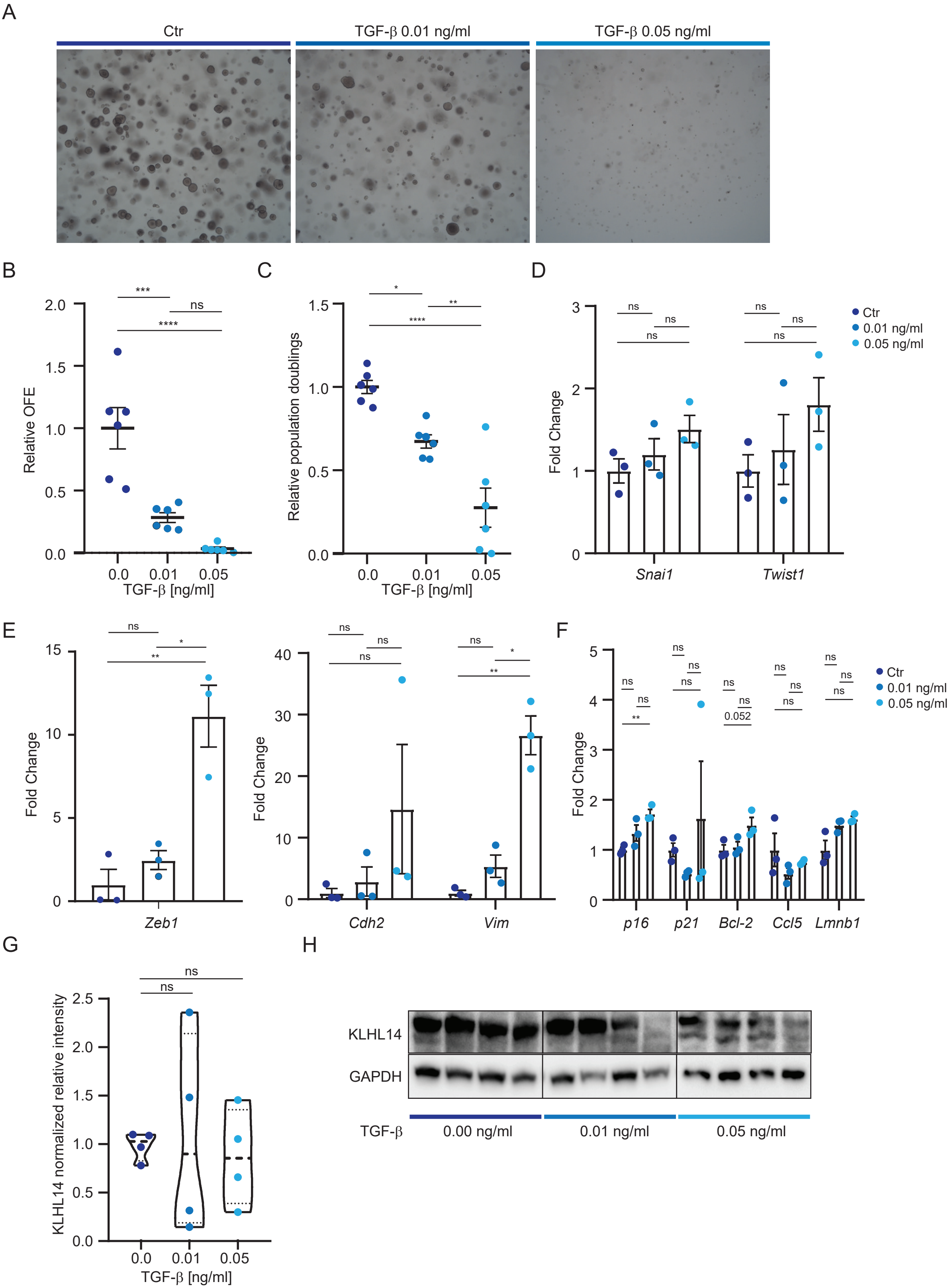
A
Ctr
TGF-β 0.01 ng/ml
TGF-β 0.05 ng/ml
B
Relative OFE
TGF-β [ng/ml]
0.0
0.01
0.05
***
ns
****
C
Relative population doublings
TGF-β [ng/ml]
*
**
****
D
Fold Change
Snai1
Twist1
ns
Ctr
0.01 ng/ml
0.05 ng/ml
E
Fold Change
Zeb1
Cdh2
Vim
ns
*
**
F
Fold Change
p16
p21
Bcl-2
Ccl5
Lmnb1
ns
**
0.052
Ctr
0.01 ng/ml
0.05 ng/ml
G
KLHL14 normalized relative intensity
TGF-β [ng/ml]
0.0
0.01
0.05
ns
H
KLHL14
GAPDH
TGF-β
0.00 ng/ml
0.01 ng/ml
0.05 ng/ml
