# Supplementary material for: Role of Klhl14 in senescence and epithelial-to-mesenchymal transition via TGF-β modulation
Source: Cell Death Dis. 2026 Jun 30;17(1):813. doi: 10.1038/s41419-026-09048-7 (PMC13586328; doi:10.1038/s41419-026-09048-7)
Supplement: Supplementary file 3 [file 41419_2026_9048_MOESM3_ESM.pdf]

A

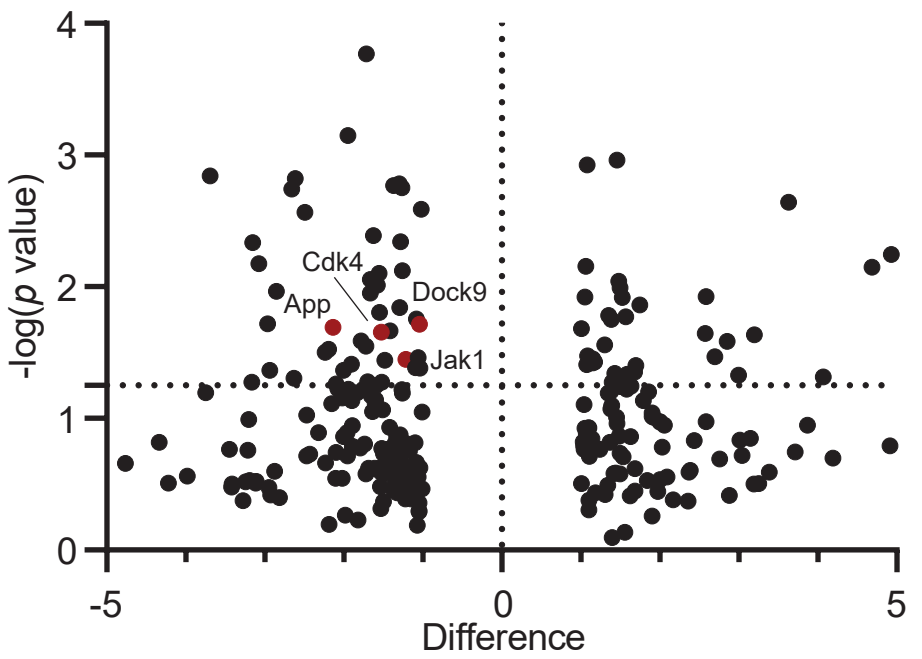

B

Input	Interacts with
App	Furin, Smad3, Smad4, Fkbp-12
Dock9	Smad2, Smad3
Cdk4	Myc, Cdkn2b
Jak1	TGF-β receptor type 2, Smad4
