# Supplementary material for: Role of Klhl14 in senescence and epithelial-to-mesenchymal transition via TGF-β modulation
Source: Cell Death Dis. 2026 Jun 30;17(1):813. doi: 10.1038/s41419-026-09048-7 (PMC13586328; doi:10.1038/s41419-026-09048-7)

Figure 2B

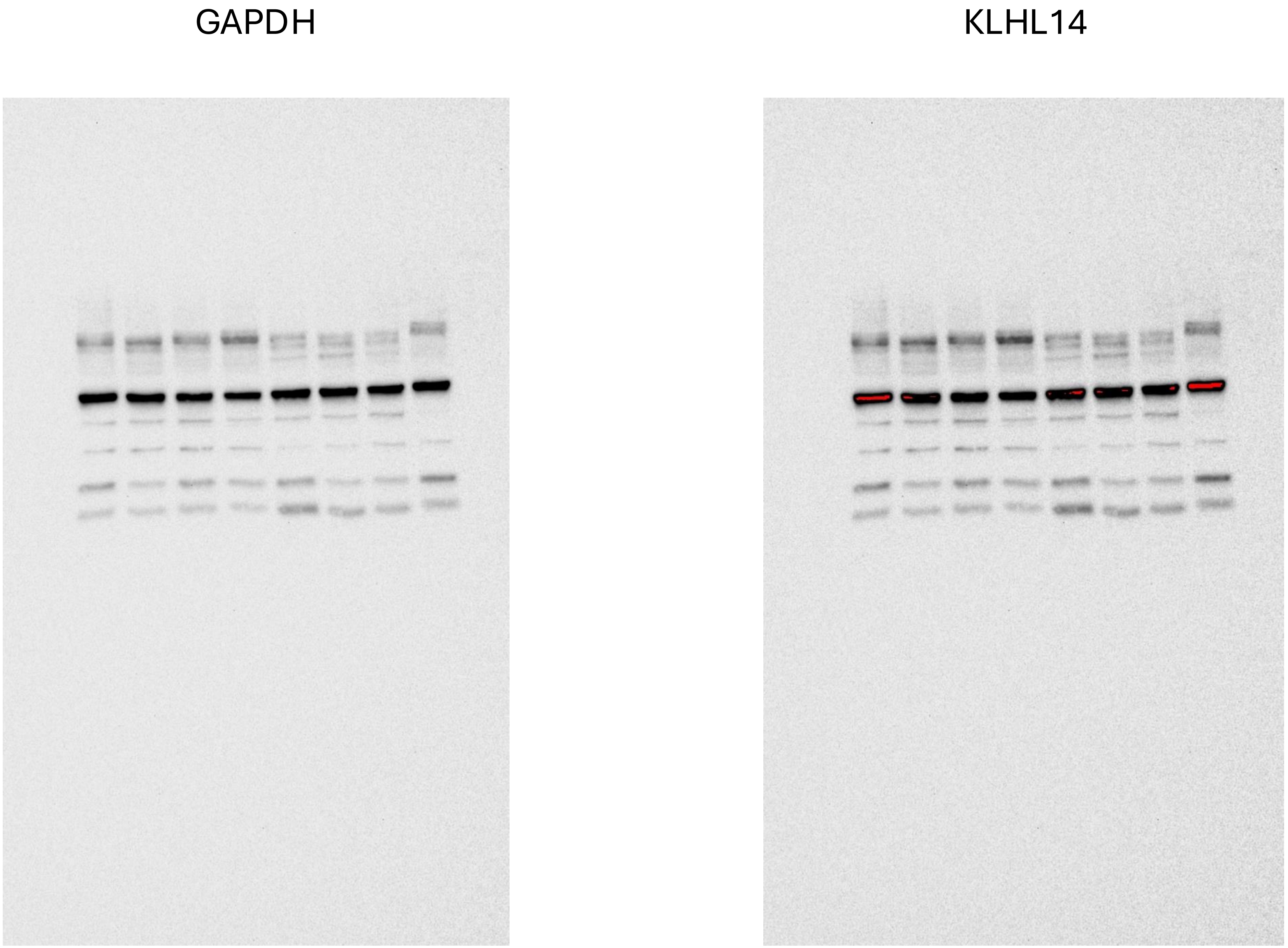

Supplementary Figure 2H

GAPDH 0.00 and 0.01 ng/ml

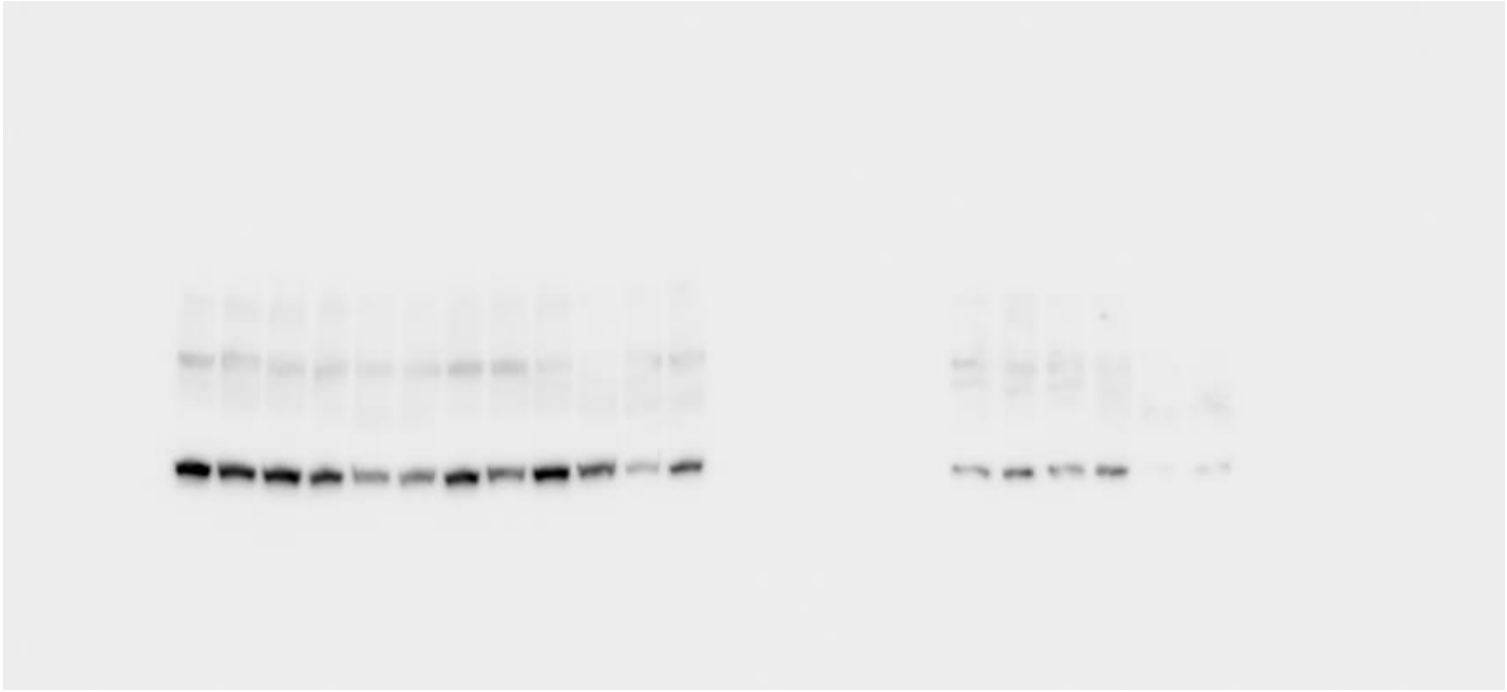

GAPDH 0.05 ng/ml

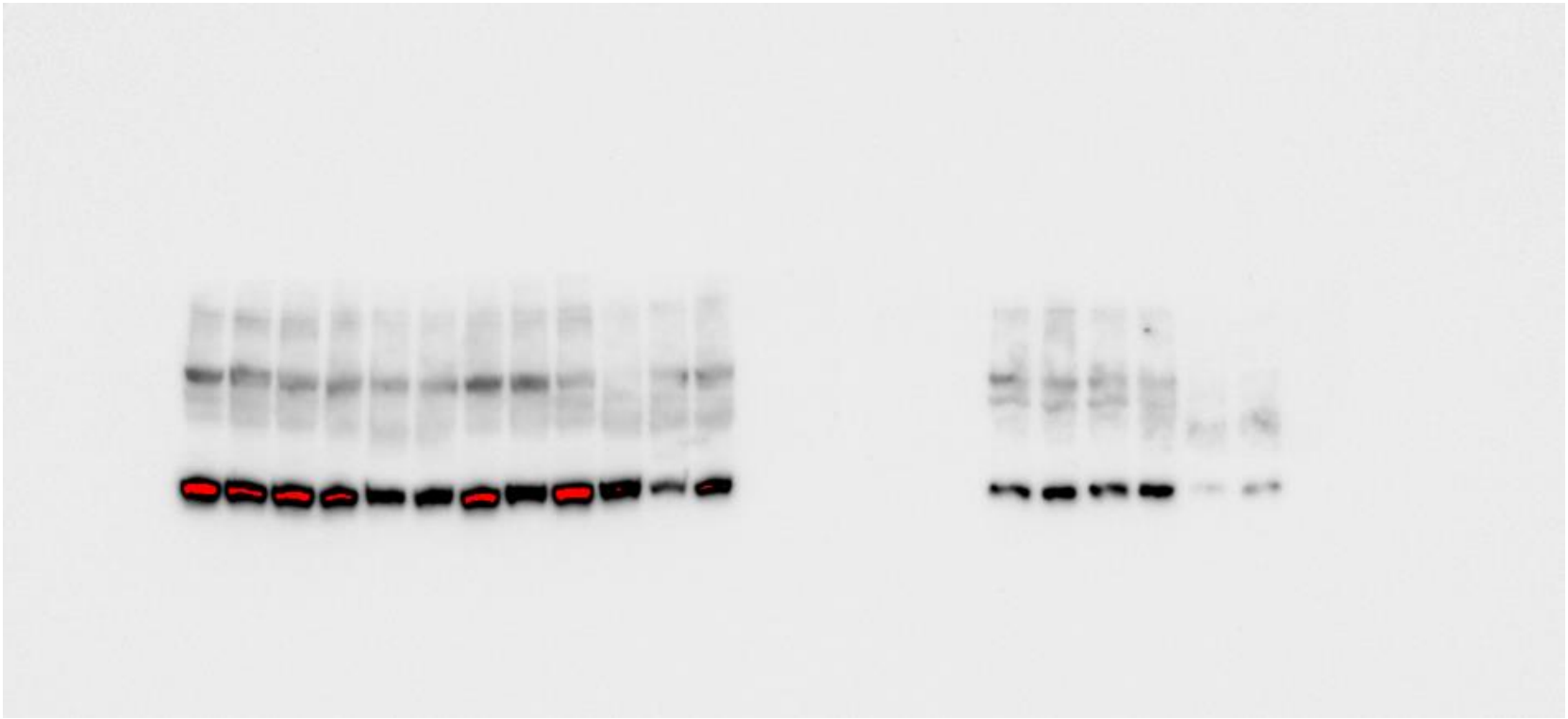

KLHL14

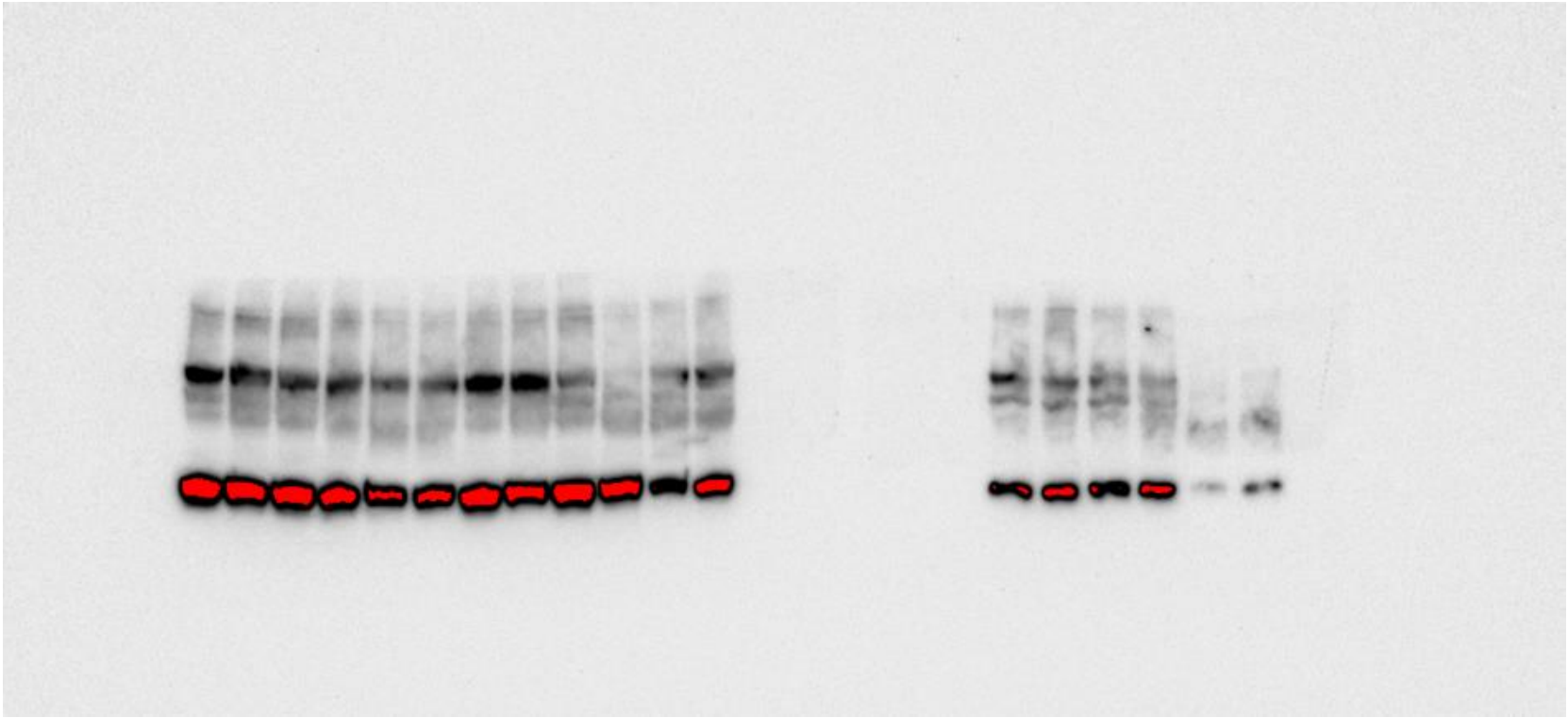

Supplement: Supplementary file 4 [file 41419_2026_9048_MOESM5_ESM.pdf]
