# Supplementary material for: Role of Klhl14 in senescence and epithelial-to-mesenchymal transition via TGF-β modulation
Source: Cell Death Dis. 2026 Jun 30;17(1):813. doi: 10.1038/s41419-026-09048-7 (PMC13586328; doi:10.1038/s41419-026-09048-7)
Supplement: Supplementary file 5 [file 41419_2026_9048_MOESM4_ESM.docx]

Supplementary Fig. 1: ***Klhl14* silencing is not linked with cell death, fibrosis, or dedifferentiation.** (A) Heatmaps displaying Z-scores of proteins part of the GSEA dataset *REACTOME_PROGRAMMED_CELL_DEATH* and (B) *WP_LUNG_FIBROSIS* retrieved from whole proteomic analysis in 7-day-old shSCRA and shKlhl14 organoids. (C) qPCR analysis on 7-day-old organoids obtained from transduced cells evaluating the expression of thyroid maturation and differentiation genes. Data are shown as mean ± SEM. Group differences were evaluated with two-tailed Student’s *t*-test. N=4. **p* < 0.05, ***p* < 0.01

Supplementary Fig. 2: **TGF-β treatments block mTGO growth.** (A) Light microscope representative pictures of 7-day-old mTGOs treated with increasing doses of TGF-β, 4x objective. (B) Relative OFE calculated on 7-day-old mTGO treated with increasing doses of TGF-β. (C) Relative population doublings calculated on 7-day-old mTGO treated with increasing doses of TGF-β. (D) qPCR analysis on 7-day-old organoids treated with increasing doses of TGF-β, evaluating the expression levels of *Snai1* and *Twist1*; (E) *Zeb1*, *Cdh2*, *Vim*; (F) senescence-related markers. (G) Normalized relative intensity of KLHL14 western blot bands reflecting the protein levels in 7-day-old organoids after TGF-β treatment. All the bands in KLHL14 frame have been considered. (H) Western blot analysis of 7-day-old organoids grown in presence of TGF-β. Data are shown as mean ± SEM. Group differences were evaluated with one-way ANOVA with post-hoc Tukey’s HSD or two-tailed Student’s *t*-test. N=3-6. **p* < 0.05, ***p* < 0.01, ****p* < 0.001, *****p* <0.0001

Supplementary Fig. 3: **Discovery-based proteomic analysis of KLHL14 interactors.** (A) Discovery-based proteomic analysis on immunoprecipitated samples to identify novel interactors of KLHL14 was analyzed to discriminate false positives. The volcano plot displays (top left quadrant) KLHL14-specific interactors, highlighting in red the proteins that bind to components of the TGF-β receptor signaling, resulted by Reactome investigation. (B) Reactome-based analysis of immunoprecipitation proteomics delineated the binding relationships between KLHL14 interactors and TGF-β signaling proteins. N=4. -log(*p* value) threshold: 1.25.

| Gene | Primers |
| --- | --- |
| Ywhaz | F: TTACTTGGCCGAGGTTGCT  R: TGCTGTGACTGGTCCACAAT |
| p21 | F: AACATCTCAGGGCCGAAA  R: TGCGCTTGGAGTGATAGAAA |
| p16 | F: GAACTCTTTCGGTCGTACCC  R: CGAATCTGCACCGTAGTTGA |
| Bcl-2 | F: GTGGCCTTCTTTGAG  R: GCCTCCGTTATCCTG |
| Mcp1 | F: GCTCAGCCAGATGCAGTTAA  R: TCTTGAGCTTGGTGACAAAAACT |
| Ccl5 | F: TGCCCACGTCAAGGAGTATTTC  R: AACCCACTTCTTCTCTGGGTTG |
| Klhl14 | F: GGCTCCTCTCCACTCACTTTC  R: TCAGCTCAGCAGCGAAGTC |
| Lmnb1 | F: GGGAAGTTTATTCGCTTGAAGA  R: ATCTCCCAGCCTCCCATT |
| Cdh1 | F: CGTCCTGCCAATCCTGATGA  R: ACCACTGCCCTCGTAATCGAAC |
| Cdh2 | F: ACAGTGGAGCTCTACAAAGG  R: CTGAGATGGGGTTGATAATG |
| Vim | F: GAACCTCCAGGAGGCCGAGG  R: CATCTTAACATTGAGCAGATC |
| Twist | F: CCAGCTCCAGAGTCTCTAGA  R: GCCAGGTACATCGACTTCCT |
| Zeb1 | F: GCAGAAAATGAGCAAAACCATGA  R: TGGGTTCTGTATGCAAAGGTG |
| Snai1 | F: ATGCACATCCGAAGCCACA  R: GACTCTTGGTGCTTGTGGAG |
| CD24 | F: GTTGCACCGTTTCCCGGTAA  R: CCCCTCTGGTGGTAGCGTTA |
| CD44 | F: TCGATTTGAATGTAACCTGCCG  R: CAGTCCGGGAGATACTGTAGC |
| EpCAM | F: GCGGCTCAGAGAGACTGTG  R: CCAAGCATTTAGACGCCAGTTT |
| Hhex | F: CGGACGGTGAACGACTACAC  R: CTTCTCCAGCTCGACGGTC |
| Bmp4 | F: ATTCCTGGTAACCGAATGCTG  R: CCGGTCTCAGGTATCAAACTAGC |
| Ocln | F: TTCCTGGTAACCGAATGCTGA  R: CCGGATAAAAAGAGTACGCTGG |
| Pax8 | F: GAGGTGAGGTGAGGTGTGGTGTGC  R: GGTTTCCTGCGATTCTGC |
| Nkx2-1 | F: CGCCTTACCAGGACACCAT  R: CCCATGCCACTCATATTCAT |

Supplementary Table 1: **Primers list for qPCR analysis.**
